# Stimulating soil microorganisms for mineralizing the herbicide isoproturon by means of microbial electroremediating cells

**DOI:** 10.1111/1751-7915.12351

**Published:** 2016-02-16

**Authors:** Jose Rodrigo Quejigo, Ulrike Dörfler, Reiner Schroll, Abraham Esteve‐Núñez

**Affiliations:** ^1^ University of AlcaláAlcalá de HenaresMadridSpain; ^2^ Helmholtz Zentrum MünchenMúnichGermany; ^3^ IMDEA‐WATER Parque Tecnológico de AlcaláMadridSpain

## Abstract

The absence of suitable terminal electron acceptors (TEA) in soil might limit the oxidative metabolism of environmental microbial populations. Microbial electroremediating cells (MERCs) consist in a variety of bioelectrochemical devices that aim to overcome electron acceptor limitation and maximize metabolic oxidation with the purpose of enhancing the biodegradation of a pollutant in the environment. The objective of this work was to use MERCs principles for stimulating soil bacteria to achieve the complete biodegradation of the herbicide ^14^C‐isoproturon (IPU) to ^14^ CO_2_ in soils. Our study concludes that using electrodes at a positive potential [+600 mV (versus Ag/AgCl)] enhanced the mineralization by 20‐fold respect the electrode‐free control. We also report an overall profile of the ^14^C‐IPU metabolites and a ^14^C mass balance in response to the different treatments. The remarkable impact of electrodes on the microbial activity of natural communities suggests a promising future for this emerging environmental technology that we propose to name bioelectroventing.

## Introduction

Biodegradation based on metabolic activities of microorganisms is the primary mechanism for pollutant removal in the environment. However, the process is highly dependent on the existence of indigenous degrading species as well as several abiotic factors (Vogt and Richnow, 2014). The absence of terminal electron acceptors (TEA) in soil might be responsible of the limited biodegradation of organic pollutants under anaerobic and strong reductive conditions (Megharaj *et al*., 2011) limiting the *in situ* microbial respiration. The supply of additional electron acceptors like oxygen (bioventing) (Kabelitz *et al*., 2009; García *et al*., 2010), humic acids (Lovley, 2000) or nitrates (Yu *et al*., 2013) to stimulate the microbial metabolism has been a common practice to remove organic pollutants, but this incurs in extra cost and specially in the case of nitrates causes secondary pollution concerns (Pandey and Fulekar, 2012).

Such a TEA limitation could be overcome using solid conductive electron acceptors like the electrodes used in microbial electroremediating cells (MERCs). MERCs are indeed variants of sediment microbial fuel cells (sMFCs). These bioelectrochemical devices use sediment‐buried electrodes (anodes) acting as electron sink coupled to the microbial oxidation of organic matter. The anode is connected through an external resistance to a cathode where electrons are finally consumed by an electron acceptor as oxygen (Tender *et al*., 2002; Venkata Mohan *et al*., 2009; Domínguez‐Garay *et al*., 2013). Both MERCs and sMFCs are bioelectrochemical devices but they differ in the operation mode. While sMFCs aim to maximize the power generation (Watts), MERCs aim to reach maximum current production (Amperes) through maximizing metabolic oxidation of organic/inorganic soil compounds (Rodrigo *et al*., 2014). In an experimental modus operandi, this is achieved configuring the MERCs with external resistances close to the short circuit or setting potentials electrode that favour the current production. So, regarding to the power curve of a bioelectrochemical device, the sMFCs work under an external resistance that allows performing at maximum power and MERCs work close to short circuit where the power is almost zero, offering an alternative to solve the constraints of electron acceptors and favouring the degradation of contaminants under soil‐flooded conditions.

Electroactive microorganisms have been shown for almost a decade to interchange electrons with conductive materials (electrodes) buried in the soil and sediments (Reimers *et al*., 2001; Tender *et al*., 2008; Domínguez‐Garay *et al*., 2013; Li and Yu, 2015). Although energy harvesting was the primary target, we have now explored additional scenarios where oxidative metabolism, e.g. mineralization of ^14^C‐isoproturon (^14^C‐IPU), can be enhanced and subsequently used as new tools for stimulating environmental bacteria. Zhang *et al*. (2010) demonstrated for the first time that graphite electrodes could serve as an electron acceptor for the degradation of toluene and benzene in polluted slurries. Since then, enhanced biodegradation of PAHs (Morris and Jin, 2012; Yan *et al*., 2012; Rodrigo *et al*., 2014; Sherafatmand and Ng, 2015), phenol (Huang *et al*., 2011), pesticides (Cao *et al*., 2015) and chlorinated organics (Chun *et al*., 2013) has been reported. It is important to point out that pollutant removal by bioelectrochemical‐assisted tools as MERCs involves biodegradation and not just the migration of contaminants (e.g. organochlorines) as classical physicochemical soil electroremediation does (Gomes *et al*., 2012).

The redox gradient in MERCs is established spontaneously across the soil–water interphase as a result of spatially segmented reduction–oxidation reactions (Li and Yu, 2015) establishing an electron transport route between electrodes. However, the soil‐buried electrode potential is typically negative as a response to the biochemical environment around it so it can be inappropriate to drive the transformation for many recalcitrant organics (Zhao *et al*., 2006). Alternatively, an external voltage can be applied between anode and cathode for stimulating bacteria activity and consequently pollutant removal (Aulenta *et al*., 2007; Chun *et al*., 2013). However, those studies just set a constant voltage value between electrodes, but no control over the anode (the electron sink electrode) was performed.

A more advanced strategy for coping with the electron redox unbalance would be to set up electrodes at a positive potential so microorganisms may have a more redox favourable TEA for performing the oxidative reactions. Actually, it has been reported an increase in the oxidative metabolism of *Geobacter sulfurreducens* by increasing electrode potentials to values as high as 600 mV (versus Ag/AgCl) (Busalmen *et al*., 2008). Then the electrodes not only overcome the TEA limitation but also allow the controllability of the bioremediation processes that can be monitored and regulated by tuning electrochemical parameters.

The absence of suitable TEAs in anaerobic and strong reductive environments like flooded soils might be responsible of the limited biodegradation of a phenylurea herbicide as isoproturon (IPU) (Larsen and Aamand, 2001). As a consequence, the presence of IPU in groundwater may exceed the approved critical value for drinking water (0.1 μg l^−1^) set by the European Community Drinking Water Directive (Folberth *et al*., 2009).

The aim of our work was to use the mineralization of ^14^C‐IPU in soil as a proof of concept to demonstrate how setting electrodes at positive potentials can have a high impact in the microbial oxidation activity. We have proved that the presence of electrodes artificially polarized at potentials as high as 600 mV (versus Ag/AgCl) stimulate the biodegradation in flooded environments. A similar effect is achieved when oxygen (800 mV) is artificially supplied in bioventing treatments. So thus, we propose the term *bioelectroventing* for referring to the process of enhancing bioremediation under soil‐flooded conditions by using electrodes as microbial electron sink.

## Results and discussion

### Enhanced isoproturon mineralization by using positive electrode potential

**Figure 1:**
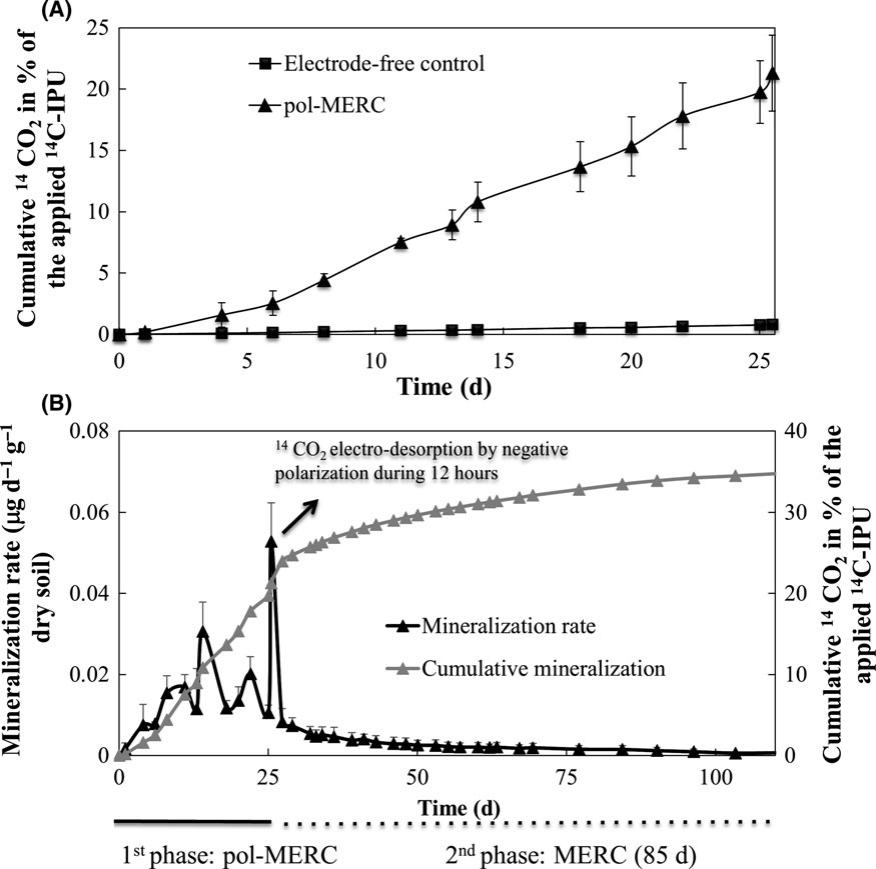
A. Cumulative mineralization of ^14^C‐IPU within 25 days in pol‐MERCs (*n* = 4, SD) compared with mineralization capability of the soil under the electrode‐free conditions (*n* = 3, SD). B. Cumulative mineralization and rate of ^14^ CO_2_ production from ^14^C‐IPU under long‐term MERCs treatment. During the first phase, the polarized anode acted as TEA at 0.6 V versus Ag/AgCl [+197 mV versus normal hydrogen electrode (NHE)]. This phase ended with the anode potential reversed to −300 mV versus Ag/AgCl for 12 h. During the second phase, the anode potential was achieved by connecting the electrodes through a 56 ohm resistor.

The first series of experiments started by investigating the influence of a positive potential electrode on the ^14^C‐IPU mineralization in soil. After the incubation period (25 days), the cumulative mineralization reached the value of 21.3% (average value of four experimental replicates), whereas ^14^C‐IPU mineralization in the electrode‐free control was below 1% (Fig. 1A). Regarding the low proportion of radiochemical impurities of the ^14^C‐IPU applied in this series (98% purity), most of the ^14^CO_2_ derives from the radiochemical pollutant. Sterile pol‐MERCs were assayed in order to prove that polarized electrodes by themselves were not performing abiotic electro‐oxidation of ^14^C‐IPU. Actually, the sterilized system (1.49 ± 0.05% mineralization after 25 days of incubation) confirms the mineralization of ^14^C‐IPU by means of the native microbial population in our soil assays. Interestingly, the cumulative mineralization under both, electrode‐free control and sterile pol‐MERCs were lower than the impurities fraction in our ^14^C‐IPU (2%).

So, the enhancing effect of the anode in the microbial metabolic processes raised the mineralization over 20‐fold in comparison with the electrode‐free control under flooded conditions (1%). Moreover, the anodic enhancement outperformed the aerobic mineralization (8%) previously reported by Folberth *et al*., 2009 using the same soil Aric Anthrosol. So thus, this fact underlines the doubtful implication of oxygen as a possible mechanism to enhance the mineralization of IPU under the presence of a polarized electrode, which is consistent with the strong reductive scenario‐taking place under soil‐flooded conditions.

Our pol‐MERCs assays achieved a high cumulative mineralization together with a high mineralization rate (Fig. 1B), both common features present in biodegradation when the pollutant acts as a source of energy for the microbial degrading community (Grundmann *et al*., 2011). In consequence, our electrode‐assisted treatment offers a malleable TEA capable to adapt to different redox‐dependent processes and perform *in situ* bioremediation by avoiding chemicals consumption or soil manipulation with negative environmental consequences.

To the best of our knowledge, it is the first time that a significantly ^14^C‐IPU mineralization has been reported under soil‐flooded conditions. Most of the research regarding biodegradation of IPU has been almost fully devoted to the aerobic metabolism, and just a few reports are available for degradation of this substance under strong reductive conditions, where significant mineralization of IPU has not being reported elsewhere. Larsen *et al*. (2000) found no mineralization of IPU anaerobically in the presence of nitrate in microcosm experiments with a sandy aquifer sediment and in a subsequent study they detected no mineralization of IPU in different aquifer sediments under denitrifying, sulfate‐reducing or methanogenic conditions following incubation for 312 days at 10°C (Larsen and Aamand, 2001). The same results were obtained examining subsurface limestone samples after 250 days (Janniche *et al*., 2010).

The neutral pH of our soil (6.8) and the flooding soil conditions of our assays allow CO_2_ to dissolve in water and establish an equilibrium with carbonic acid leading to negative‐charged species as HCO_3_^−^ and CO_3_^2−^ that could be adsorbed on the surface of positive polarized electrodes. In order to evaluate this hypothesis, just after 25 days of assay, the electrode potential was shifted from positive potential (+600 mV versus a Ag/AgCl) to negative one (−300 mV versus a Ag/AgCl) in both pol‐MERCs and sterile pol‐MERCs (Fig. 1B). The negative potential was kept for 12 h to release and monitor the ^14^CO_2_ electro‐adsorbed in the anode. The mineralization rate increased in pol‐MERCs from 0.01 to 0.05 μg day^−1^ g^−1^ (dry soil) of ^14^C‐IPU mineralized but not increased was registered in sterile pol‐MERCs.

The transitory assay of reversing the electrode potential was followed by a long‐term assay for evaluating the response of the electroactive microbial community to a different electrode potential. So thus, two of the four pol‐MERCs replicates were kept running for 12 additional weeks after converting the pol‐MERCs into MERCs by substituting the potentiostat by a low external resistor (Fig. 1B). The new configuration led to a drop off in the redox anode potential from +600 mV (versus Ag/AgCl) to a negative anode potential [−200 mV (versus Ag/AgCl)] for the rest of the assay. The effect was evident from the shift in the slope for the cumulative ^14^C‐IPU mineralization, decreasing from three‐ to nine‐fold over the course of the second phase. This strongly supports the key role of setting a positive anode potential to achieve an optimal *in situ* biodegradation of IPU.

It is an accepted practice in traditional bioremediation techniques like bioventing (Kabelitz *et al*., 2009; García *et al*., 2010) to support microbial respiration by supplying a TEA with a positive redox potential like oxygen (+800 mV). One disadvantage of these strategies is the necessity to supply continuously the oxidant, which spread out to the environment and negatively affect the native microbial community. In contrast pol‐MERCs provide an endless, low priced and sustainable terminal electron sink showing minimal environmental disturbance (Logan *et al*., 2006). So thus, we propose the term *bioelectroventing* for referring to the process of enhancing bioremediation by using electrodes as microbial electron sink.

### Electrochemical performance of pol‐MERCs

**Figure 2:**
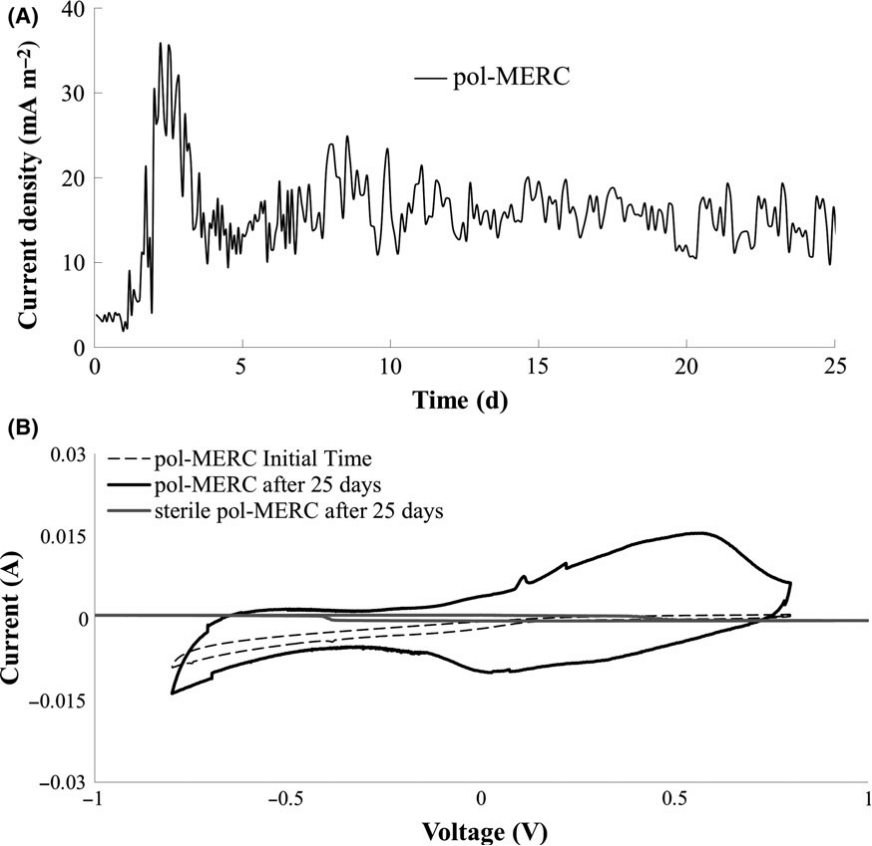
A. Chronoamperometry of pol‐MERCs polarized at 0.6 V (versus Ag/AgCl). Geometrical anode surface was used for calculating the current density. B. Cyclic voltammetry tests (scan rate: 1 mV s^−1^) carried out at initial experimental time and after 25 days under bioelectrochemical‐assisted soil (pol‐MERCs and sterile pol‐MERCs).

In order to evaluate the electrochemical performance the current production was continuously registered in pol‐MERCs (Fig. 2A). Soil under this treatment showed just 1 day of lag phase, reaching a maximum current density of 35 mA m^−2^ before entering into steady‐state (ca. 15 mA m^−2^).

To give insight into the nature of this response, we performed cyclic voltammetries (CV) of the anodes from pol‐MERCs. A different bioelectrochemical profile was observed after 25 days of incubation. In fact, Fig. 2B shows an inflexion peak centred at +0.300 V (versus Ag/AgCl reference) in comparison with initial time anodes and with the CV of the sterile pol‐MERCs. So thus, the intensity of the signals provides an indication of an enrichment of microbial communities with electron transfer capabilities.

In addition, CV analysis did not show any peak corresponding to the oxidation of water, even after performing the analysis under a wide voltage interval (from +1 V to −1 V). This issue confirmed that the electrode potential applied was not enough to drive oxygen production at the anode.

### Characterization of the ^14^C‐IPU mass balance

**Figure 3:**
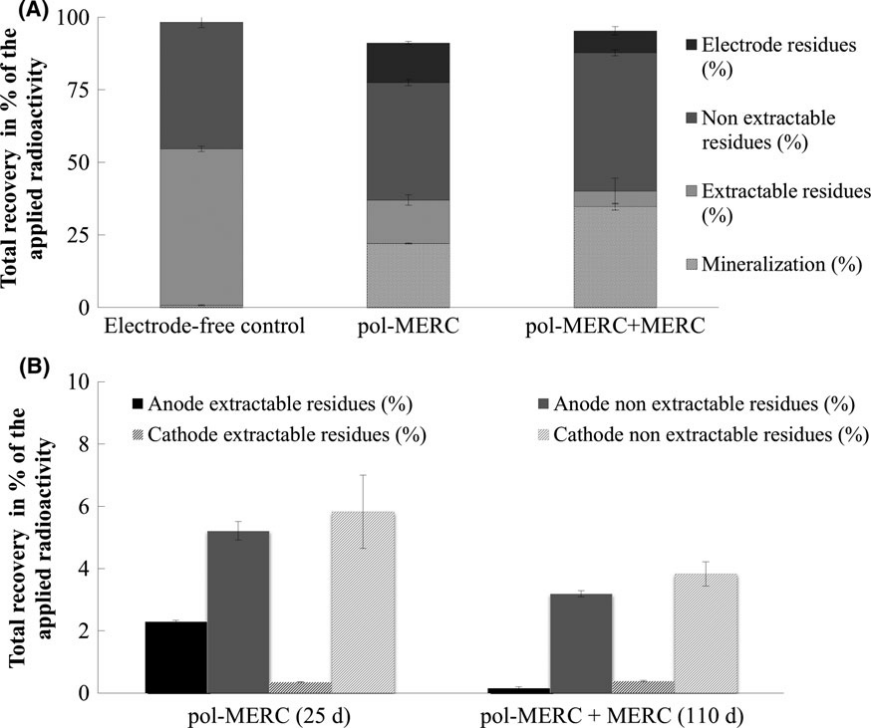
A. Distribution and mass balance of different soil treatments regarding initial radioactivity of ^14^C‐IPU. B. Extractable and non‐extractable ^14^C residues in carbon felt electrodes used under different treatments. Bars represent standard deviation.

In addition to the cumulative ^14^CO_2_ mineralization, the soil‐ and electrode‐associated radioactivity should be measured in order to conform the ^14^C mass balance established in our experimental system. So thus, methanol extractable residue (ER) and non‐extractable residues (NER) of ^14^C‐IPU that remained in the soil and electrodes during the course of the assays were shown in Fig. 3A. Interestingly, these mass balances ranged between 91.2% and 98.4% of the initially supplied ^14^C‐IPU under pol‐MERCs and electrode‐free control respectively. This parameter was therefore an indicator of the effectiveness of our experimental design and underlines the presence of potential losses of ^14^CO_2_, especially under conditions of high mineralization rates (pol‐MERCs treatment). The extractable ^14^C‐residues in the soil samples varied considerably between the different experimental conditions after 25 days of incubation. The ER did reach 53.9% of the applied radioactivity for the electrode‐free control but it did just 15% in soil under the pol‐MERCs treatment. On the contrary, a similar distribution was observed for NER regardless the treatment: 43.6% for electrode‐free control and 40.4% for pol‐MERCs. Examining the ^14^C mass balance of the long‐term assay (110 days), we observed an increase in the NER in comparison with the standard pol‐MERCs. The formation of NER are often explained by binding of a xenobiotic to the soil matrix, specially to the soil organic matter and has been reported that IPU metabolites are mostly adsorbed onto organic matter in soils (Ertli *et al*., 2004). Another possible pathway for the formation of NER is the ^14^C‐IPU‐degradation by microorganisms that can use it as an energy source and for microbial growth; as a result, ^14^C‐residues are incorporated into biomolecules and e.g. subsequently bound to soil (‘apparent NER’) when microbes die (Grundmann *et al*., 2011). Positive correlations between NER formation and IPU aerobic mineralization have been previously reported (Alletto *et al*., 2006), which could explain the similar NER fraction under electrode‐free control and pol‐MERCs despite the broad ^14^C‐IPU cumulative mineralization.

Adsorption‐desorption assays were conducted to exam the affinity of ^14^C‐IPU and different electro conductive materials. Carbon felt showed a convenient physic‐mechanical properties and it was established as the electrode material for our assays due to the lack of IPU adsorption (Figs. S1 and S2). Nevertheless, to calculate the total recoveries of ^14^C‐radioactivity at the end of the experiments we extracted by accelerated solvent extraction (ASE) and combusted the carbon felt electrodes, both the anode and the cathode separately (Fig. 3B). The total amount of ^14^C radioactivity, extractable and non‐extractable, in the electrodes reached 13.7% in pol‐MERCs. In the long‐term assay where pol‐MERCs treatment was followed by MERC treatment, the percentage of total ^14^C‐residues in the electrodes was reduced to 7.8%. Interestingly, the amount of ER (0.2%) was extremely low in those anodes that were used for such a long incubation period. A similar result was previously reported by Zhang *et al*. (2010) using polluted slurries, although they reported a higher adsorption for benzene and toluene in the electrodes (70%) and also confirmed that toluene adsorbed on the graphite could be metabolized.

### ^14^C‐IPU biodegradation pathway

Since the discovery and marketing of phenylurea herbicides shortly after the Second World War, this group of chemicals has grown to be one of the most important classes of herbicides for agricultural use (Sørensen *et al*., 2003). The removal of IPU from the environment has received considerable attention, mainly under aerobic conditions and despite many anoxic environments like groundwater aquifers, subsurface soil and seasonally flooded agricultural soils, biodegradation of IPU under anaerobic and strong reductive conditions is largely unknown (Larsen and Aamand, 2001). The absence of suitable TEA might be responsible of the limited biodegradation of IPU under strong reductive scenarios (Larsen and Aamand, 2001; Megharaj *et al*., 2011). Interestingly, this is the first time that IPU is reported to be mineralized under soil‐flooded conditions so we can conclude that the use of polarized electrodes have accelerated the finding of new biodegradation pathways that were not found for decades.

Typical mineralization pathway of IPU by bacterial strains includes two successive N‐demethylations, cleavage of both the urea side‐chain and the aromatic ring, and a final mineralization to CO_2_ and production of biomass (Sørensen and Aamand, 2001). Two alternative metabolic pathways were reported involving initial hydroxylation of the isopropyl side‐chain, resulting in either 1‐OH‐IPU, a dead‐end metabolite just detected in bacterial cultures derived from soil, or 2‐OH‐IPU, described in agricultural soils (Lehr *et al*., 1996; Scheunert and Reuter, 2000). These metabolites were reported just under aerobic pathways. Just a few reports about the anaerobic degradation of this substance are available and they do not included information about either the microbial reactions or their metabolites accumulated under flooded conditions (Larsen *et al*., 2000; Larsen and Aamand, 2001; Janniche *et al*., 2010).

Exhaustive analysis of our treated soil revealed six radioactive IPU metabolites that were compared with authentic analytical standards. In addition to the parent compound IPU, we have successfully identified three metabolites and detected two unidentified metabolites among the degradation products (Table 1): monodemethyl‐isoproturon (MDIPU), (3‐(4‐isopropylphenyl)‐1‐methylurea), didemethyl‐isoproturon (DD‐IPU) (3‐(4‐isopropylphenyl)‐urea) and 2‐OH‐mono‐demethyl‐isoproturon (2‐OH‐MIPU) 3‐(4‐(2‐hydroxyisopropylphenyl))1‐methylurea. IPU as well as any other phenylurea is not subjected to chemical degradation within the pH range of 4–10. Consequently, chemical degradation of IPU in soils is of minor importance (Sørensen *et al*., 2003) making it a recalcitrant xenobiotic. So thus, the metabolites observed in this study therefore resulted from microbial attack of the parent compound.

**Table 1:** Profile composition of the methanol extractable residues (Peaks areas appear as % of extract‐^14^C and metabolite concentrations as μg g^−1^ dry soil) for either, electrode‐free control and polarized‐MERCs

Isoproturon and metabolites	RT (min)	Electrode‐free control		pol‐MERC	
		Area %	μg g^−1^ dry soil	Area %	μg g^−1^ dry soil
2‐OH‐MIPU^a^	11.90 ± 0.11	0.72 ± 0.16	0.024 ± 0.008	3.87	0.035
Unidentified	13.01 ± 0.05	2.75 ± 0.51	0.081 ± 0.014	6.53	0.058
Unidentified	13.94 ± 0.05	1.82 ± 0.04	0.040 ± 0.009	2.76	0.025
DD‐IPU^b^	17.60 ± 0.06	1.39 ± 0.17	0.029 ± 0.005	6.52	0.058
MDIPU^c^	18.70 ± 0.02	16.82 ± 0.35	0.421 ± 0.006	60.77	0.543
IPU^d^	19.95 ± 0.02	76.5 ± 0.19	1.913 ± 0.006	19.98	0.179
4‐IPA^e^	n.d	n.d	n.d	n.d	n.d

a 3‐(4‐(2‐hydroxyisopropylphenyl)) 1‐methylurea.

b [3‐(4‐isopropylphenyl)‐urea].

c [3‐(4‐isopropylphenyl)‐1‐methylurea].

d [3‐(4‐isopropylphenyl)‐1,1‐dimethylurea].

e 4‐isopropyl‐aniline.

*n* ****=** **3 ± SD except pol‐MERC which analysis was conducted with pooled samples replicates to exceed detection limit, therefore no standard deviation can be given.

n.d: not detectable.

**Figure 4:**
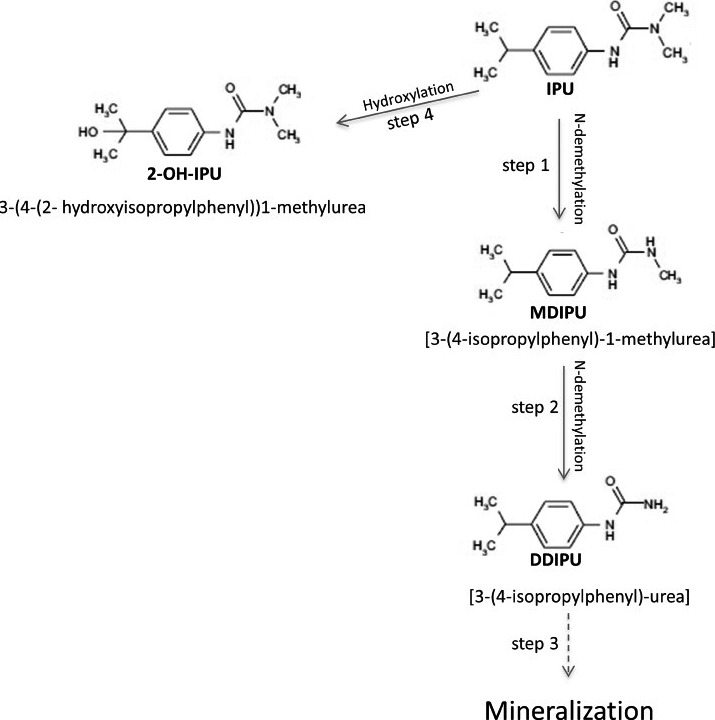
Proposed biodegradation pathways for IPU under polarized‐MERCs conditions.

The two most abundant metabolites were obtained after demethylation of the *N*,*N*‐dimethylurea side‐chain to generate MDIPU and DD‐IPU (Fig. 4, steps 1 and 2). MDIPU has been previously reported to be the most abundant metabolite after IPU biotransformation in agricultural soils, as well as in pure cultures assays by soil fungi and bacteria (Sørensen *et al*., 2003). Moreover, the abundance levels for MDIPU in pol‐MERCs extracts were ca. five‐fold higher than in environments under negative redox potential like the electrode‐free control. On the contrary, the abundance of IPU in pol‐MERCs extracts was ca. fivefold lower, going down the concentration from the initial 5 (±0.1) μg g^−1^ dry soil to 0.179 μg g^−1^ dry soil, while under electrode‐free control, the decrease reached only 1.9 (±0.006) μg g^−1^ dry soil. These concentrations were calculated based on the ^14^C chromatograms from the HPLC (coupled to a radioactivity detector) and the specific radioactivity of our standard applied in the soil (radioactivity per unit mass of the stated compound).

Monodemethyl‐isoproturon has been reported to generate 4‐IA before cleavage of the aromatic ring and final mineralization to CO_2_ (Sørensen and Aamand, 2001). So thus, low concentrations of 4‐IA have been detected in IPU‐treated agricultural soils (Lehr *et al*., 1996) and during the mineralization of IPU by *Sphingomonas* sp. strain SRS2 (Sørensen and Aamand, 2001). On the contrary, none of our bioremediation treatments showed 4‐IA among the radioactive metabolites from ^14^C‐IPU (Fig. 4). It has being reported the irreversible bound of this metabolite to soil organic matter, increasing the NER and subsequently decreasing the availability of the compound to the degrading microbial communities (Scheunert and Reuter, 2000; Johannesen *et al*., 2003). The hydroxyl derivative 2‐OH‐MIPU in our experiments has been detected at levels ca. fivefold higher in pol‐MERCs extracts than under electrode‐free control (Fig. 4, step 4). This intermediate is part of an alternative metabolic path initiated with the hydroxylation of the isopropyl side‐chain. Regarding the unidentified metabolites, they were only observed in low levels and just slight changes were observed when the electrode was set at positive potential, suggesting that they are due to biological reactions from the soil.

Although oxygen cannot be generated under our conditions, our assays revealed common metabolite patterns with aerobic conditions. Interestingly burying a positive potential electrode in flooded soil may remodel the redox scenario and as a consequence may influence the dominant energy metabolic pathways leading to common metabolite patterns with aerobic conditions. This new redox context under the influence of the electric field of the polarized electrode may change the permeability of cell membrane, leading to the excessive absorbance of extracellular substances and further change the microbial metabolism (Rittmann and McCarty, 2001). In addition, predominance of anode respiration over fermentation (Hunt *et al*., 2010; Pinchuk *et al*., 2011) and a suppression of several anaerobic species, such as archaea and sulfide‐producing populations at high redox potentials have been recently reported (Lu *et al*., 2014a; Ueno and Kitajima, 2014). Interestingly, bioenergetics and therefore the profile of metabolites during IPU biodegradation may change by shifting from substrate‐level phosphorylation (i.e. anaerobic fermentation) to oxidative phosphorylation (i.e. electrode respiration).

Further research is currently being performed to unveil whether these processes or some others as electro osmotic flow inside the saturated soil could be affected by the polarized electrode presence. Moreover, it is still unclear how a polarized electrode affects the microbial community activity to enhance *in situ* bioremediation.

## Experimental procedures

### Chemicals

Uniformly ^14^C ring‐labelled isoproturon [3‐(4‐isopropylphenyl)‐1,1‐dimethylurea] (^14^C‐IPU) with a specific radioactivity of 9.96 kBq μg^−1^ and radiochemical purity of 98% according to the producer was purchased from GE Healthcare (Little Chalfont, UK) and used as a representative pesticide for phenylurea herbicides. The ^14^C‐IPU was mixed with unlabelled IPU to provide a final concentration of 1.75 mg ml^−1^ and a specific radioactivity of 154 Bq μg^−1^. The new mix was denominated ‘^14^C‐IPU standard mix’. Non‐labelled IPU, MDIPU [3‐(4‐isopropylphenyl)‐1‐methylurea], 2‐OH‐MIPU 3‐(4‐(2‐hydroxyisopropylphenyl))1‐methylurea, DD‐IPU [3‐(4‐isopropylphenyl)‐urea] and 4‐isopropyl‐aniline (4‐IPA) were purchased from Dr. Ehrenstorfer (Augsburg, Germany; purity 99.5%). Scintillation cocktails (Ultima Gold XR and Ultima Flo AF) were obtained from Packard (Dreieich, Germany). All other chemicals and solvents were of analytical grade and were purchased from Merck (Darmstadt, Germany).

### Soil

The soil material was an Aric Anthrosol from an agricultural field (Hohenwart; latitude: 48.250, longitude: 11.567, elevation 472 m) in Germany without IPU history and with an organic matter content of 0.99%. A complete physical–chemical analysis of this soil was previously reported by Grundmann *et al*. (2011). Soil samples were taken from 0 to 20 cm and were stored in plastic bags at −20°C according to guidelines of the Organization for Economic Cooperation and Development (Organisation for Economic Cooperationand Development, 1995). The soil samples were unfrozen 2 weeks before the start of the experiment following the next incubation protocol: first they were kept at +4°C for 7 days, and then another 7 days at room temperature (20 ± 1°C). The first day at room temperature the soil was moistened to a water potential close but below −15 kPa and compacted to a density of 1.3 g cm^−3^ to equilibrate all microbial processes and ensure comparable conditions at the start of the experiments. Previous studies have shown that the microbial activity is at its optimum at these soil conditions (Schroll *et al*., 2006). The conductivity, 0.247 mS cm^−1^, was measured just before the set up of the experiment with the commercially available ECa sensors UMP‐1 (Umwelt‐Geräte‐Technik, Freising/Weihenstephan, Germany). This tool can be used for *in situ* EC measurements with minimal disruption because the sole requirement consists in burying the probe needs in the soil. The measurements were conducted under the same conditions (water content and temperature) than for the biodegradation assay.

### Spiking of the soil samples

Exactly 100 μl of ^14^C‐IPU standard mix was applied to an aliquot of 3 g dried‐and‐ground soil and homogeneously mixed. After evaporation of the organic solvent (methanol), the soil aliquot was mixed with 32 g (dry weight equivalent) of equilibrated soil with the goal to distribute the pollutant homogenously resulting in a concentration of 5 (±0.1) μg g^−1^ soil (dry weight) and a radioactivity of 154 Bq μg^−1^. The spiked soil sample was transferred to the opaque glass flask of the laboratory system described below, compacted to a soil density of 1.3 g cm^−3^ and adjusted to flooded conditions (water holding capacity + 35 ml extra deionized water). As a result 2 cm water body was maintained above the soil in order to ensure flooded conditions. Water evaporation was compensated three times per week by the addition of deionized water.

### Laboratory system

The mineralization experiment was conducted in a laboratory system built in approximation to the OECD guideline for testing of chemicals 304A (Organisation for Economic Cooperation and Development, 1981). It consisted of opaque glass flasks (250 ml volume; neoLab, Heidelberg, Germany), which were closed with a rubber stopper (neoLab); at the bottom of the stopper a plastic beaker of 25 ml volume (VWR International, Darmstadt, Germany) was attached. The plastic beaker was filled with 10 ml of 0.1 N NaOH (Merck) to trap the ^14^CO_2_ resulting from the mineralization of ^14^C‐IPU. The NaOH solution was exchanged three times per week and from the collected NaOH solution an aliquot of 2 ml was taken, mixed with 3 ml Ultima FLO AF (PerkinElmer, Rodgau, Germany) and the radioactivity was measured in a liquid scintillation counter (Tricarb 1900 TR; Packard). A hollow needle (neoLab) conducted through the rubber stopper allowed a constant supply of O_2_. To prevent saturation of the NaOH solution with atmospheric CO_2_, a plastic reservoir (neoLab) filled with soda lime (Merck) was connected to the needle.

### MERCs: Operating conditions

**Figure 5:**
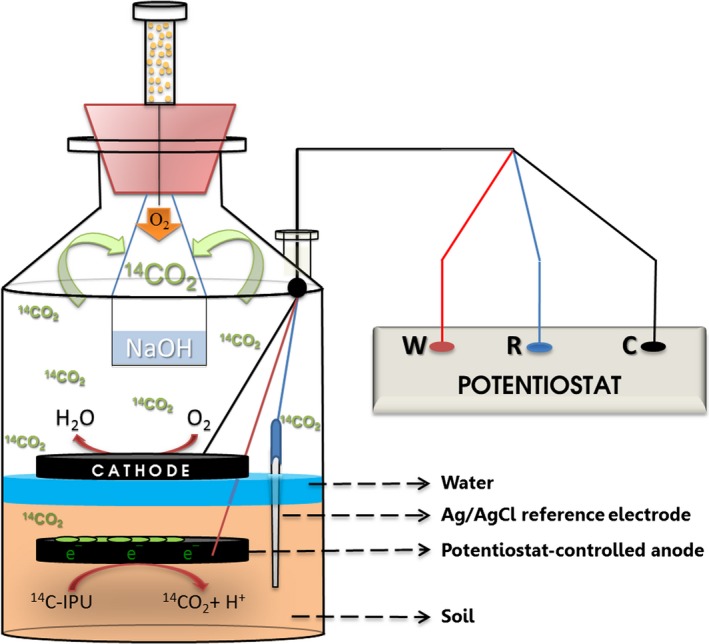
Scheme of a pol‐MERC. The anode was polarized at 0.6 V versus Ag/AgCl [+197 mV versus normal hydrogen electrode (NHE)]. ^14^ CO_2_ was trapped in a 0.1 N NaOH solution for allowing the measurement of ^14^C‐IPU.

Microbial electroremediating cells were assembled in the laboratory system (Fig. 5). The electrode used in this experiment was carbon felt (Sofacel, Barcelona, Spain), as it showed no IPU adsorption (Figs. S1 and S2) and very adequate mechanical properties to conform the system. Although a diversity of electrode materials can serve as an electron acceptor for microbial respiration, carbon felt as graphite can provide a low‐cost, low‐maintenance, continuous sink for electrons as it does not corrode or otherwise degrade during long‐term deployments (Reimers *et al*., 2006; Tender *et al*., 2008) so it is easy to remove from the soil after the treatment, showing a low impact for the environment. The electrodes were allocated at the bottom of the soil layer (anode) and above the water body (cathode). The geometrical area of the electrodes was 39 cm^2^ (surface area: 0.7 m^2^ g^−1^).

Microbial electroremediating cells were continuously operated at a poised anode potential of +600 mV versus Ag/AgCl reference electrode (RE‐5B; BASi, West Lafayette, USA) (+199 mV versus SHE) by using a potentiostat (NEV2 nanoelectra). Henceforth, these MERCs will be designated as pol‐MERCs.

Abiotic electrochemical reactions on ^14^C‐IPU were evaluated by using sterile pol‐MERCs. The soil was sterilized with γ‐irradiation. The use of gamma γ‐irradiation as a method for soil sterilization for laboratory experiments has been recommended over other sterilization techniques (McNamara *et al*., 2003) with advantages as highly effectiveness at sterilization while avoiding chemical contamination. Moreover, small doses of γ‐irradiation are capable of sterilizing large soil samples without increasing soil temperature or pressure. Gammacell 220 cobalt‐60 irradiation unit was used to sterilize samples contained in vented polypropylene tubes for 96 h at a rate of 8.33 Gy min^−1^ for a total γ‐ray dosage of 60 KGy. Biologic activity was tested by dish‐plate inoculation. Eluates of soil were prepared using phosphate buffer (pH 8) in 1:10 soil–water ratio. 1:10 and 1:100 dilutions were carried out and incubated at 30°C in LB agar plates during 72 h. Colonies formation was detected only in plates seeding with eluates of non‐sterilized soil.

Electrode‐free controls were assembled in the laboratory system without the presence of the electrodes and under the same water content, temperature and ^14^C‐IPU concentrations than pol‐MERCs.

### Mineralization assays

The mineralization of ^14^C‐IPU to ^14^CO_2_ was studied in a closed aerated laboratory system, as described before. The soil samples were incubated at 30 ± 0.1°C for 25 days in the dark. The first sampling of the NaOH trap was performed 24 h after ^14^C‐IPU additions. Then sampling was performed three times per week.

The ^14^CO_2_ production rate was expressed as μg day^−1^ g^−1^ of ^14^C‐IPU and the cumulative ^14^CO_2_ as percentage of the applied ^14^C‐IPU. After 25 days of incubation, two of the four replicates of the pol‐MERCs treatment were sacrificed for soil analysis. The other two replicates were shifted into MERCs conditions where anode and cathode were connected by a copper wire using a 56 Ω external resistor (R), where the redox potential of the anode in MERCs was set by the redox potential differences across sediment/water. ^14^C‐IPU mineralization was monitored for another 12 weeks in the absence of artificial anode poisoning.

### ^14^C‐IPU residue analysis

At the end of the incubation periods, all soil samples were extracted, and the extracts were cleaned up and analysed by HPLC. The ^14^C NERs were quantified by combustion.

Soil aliquots and carbon felt were separately extracted with methanol in an accelerated solvent extractor (ASE 200; Dionex, Idstein, Germany) at 90°C, with a pressure of 10 MPa. One hundred microlitre aliquots of each extract were mixed with 5 ml Ultima Gold XR and measured by liquid scintillation counting. The ASE extracts were concentrated on a rotary evaporator to remove the organic solvent. The concentrated extracts were adjusted to 250 ml with distilled water and subjected to solid phase extraction (SPE; Lichrolut ENV 200 mg; Varian, Darmstadt, Germany). After extraction, the SPE columns were dried under a gentle nitrogen stream and eluted with 10 ml methanol. The eluate was concentrated to a volume of 1 ml with a rotary evaporator and further concentrated under a gentle nitrogen stream to volumes between 40 and 380 μl, depending on the total ^14^C‐radioactivity of the samples. The samples were immediately analysed by HPLC. Twenty microlitre of each of these samples were injected to a HPLC system, consisting of a L‐6200 Intelligent Pump (Merck‐Hitachi, Darmstadt, Germany) a UV/VIS detector (240 nm; Merck) and a radioactivity detector LB 506 C1 (Berthold, Wildbad, Germany). The column used was a Lichrospher 100 RP‐18, 5 μm, 4 × 250 mm (Merck). The mobile phase consisted of A = acetonitrile (HPLC grade) and B = water (Lichrosolv water for chromatography; Merck) at a flow rate of 1 ml min^−1^. The gradient programme was as follows: T0 min 95% A, T15 min 40% A, T20 min 40% A, T25 min 95% A, T30 min 95% A. Parent compound and metabolites were identified by comparison of their retention times with reference substances.

After performing ASE, in order to achieve the quantification of ^14^C‐labelled NER, soil material was homogenized intensively before combusting. Three aliquots (each approximately 250 mg) of each soil sample were filled into combustion cups and mixed with 7–8 drops of saturated aqueous sugar solution to guarantee a complete oxidation of the ^14^C. Carbon felt electrodes from each assay were cut in pieces and placed into combustion cups. Combustion was conducted with an automatic sample‐oxidizer 306 (Packard). ^14^CO_2_ was trapped in Carbo‐ Sorb E (Packard) and mixed with Permafluor E (Packard) prior to scintillation counting.

### Electrochemical analysis

The anode potential in pol‐MERCs was continuously poised at +600 mV versus Ag/AgCl (+197 mV versus normal hydrogen electrode) reference electrode (RE‐5B; BASi) using a potentiostat (NEV1‐3 Nanoelectra, Madrid, Spain) and the resulting current was recorded every 60 s.

Cyclic voltammetry was recorded to qualitatively characterize the electrochemical activity of the soil by imposing at a scan rate of 1 mV s^−1^ from the open circuit voltage potential by a potentiostat (NEV3 Nanoelectra).

## Supporting information


**Fig. S1.** ^14^C‐IPU adsorption in different electro‐conductive materials.

**Fig. S2.** ^14^C‐IPU desorption in graphite paper.
