# Supplementary figures and images for: Stimulating soil microorganisms for mineralizing the herbicide isoproturon by means of microbial electroremediating cells
Source: Microb Biotechnol. 2016 Feb 16;9(3):369–80. doi: 10.1111/1751-7915.12351 (PMC4835573; doi:10.1111/1751-7915.12351)

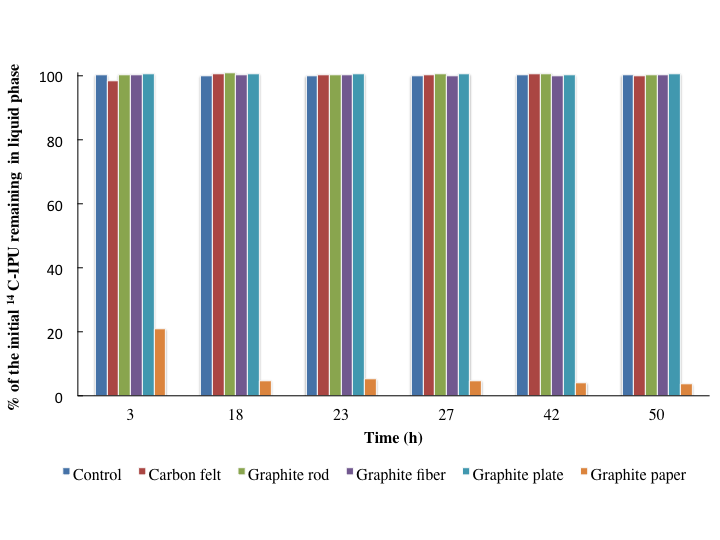

Supplement: Supplementary file 1 — Fig. S1. 14C‐IPU adsorption in different electro‐conductive materials. [file MBT2-9-369-s001.tiff]

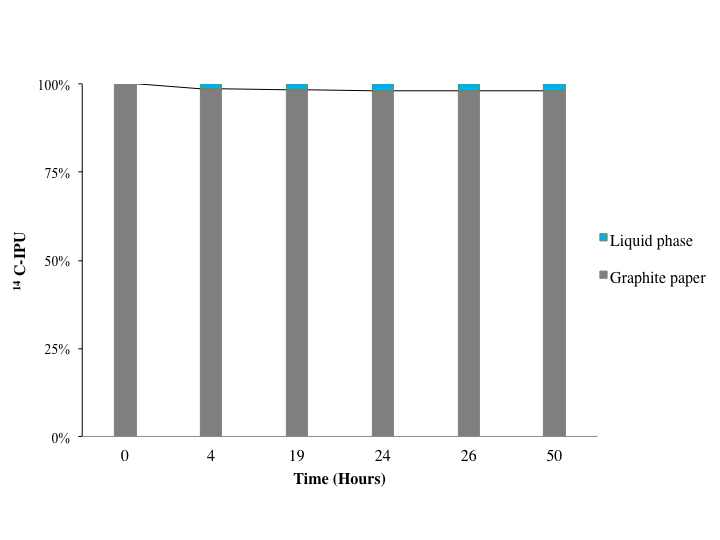

Supplement: Supplementary file 2 — Fig. S2. 14C‐IPU desorption in graphite paper. [file MBT2-9-369-s002.tiff]
